# Hydroxychloroquine in the treatment of sarcoidosis-associated uveitis and idiopathic uveitis

**DOI:** 10.1186/s12348-026-00598-7

**Published:** 2026-05-28

**Authors:** Sarah Plavonil, Robin Jacquot, Arthur Bert, Yvan Jamilloux, Laurent Kodjikian, Pascal Seve, Thomas El-Jammal

**Affiliations:** 1 Department of Internal Medicine, University Hospital of Martinique, CS 90632, Fort-De-France Cedex, Martinique; 2 Université des Antilles, Pointe-à-Pitre, 97110 Guadeloupe; 3 Department of Internal Medicine, University Hospital Lyon Croix-Rousse, Lyon, 69004 France; 4 Department of Ophthalmology, University Hospital Lyon Croix-Rousse, Lyon, 69004 France; 5 Université Claude Bernard Lyon 1, Villeurbanne, 69100 France; 6 Laboratoire MATEIS, UMR-CNRS 5510, INSA, Université Lyon 1, Villeurbanne, 69100 France; 7 Research on Healthcare Performance (RESHAPE), INSERM U1290, Université Claude Bernard Lyon 1, Lyon, France

**Keywords:** Sarcoid uveitis, Hydroxychloroquine, Idiopathic uveitis

## Abstract

**Background:**

Uveitis is a major cause of blindness in developed countries, and adverse effects associated with long-term use of topical or systemic steroids and immunosuppressive agents are notable. This study aimed to evaluate the efficacy and safety of hydroxychloroquine (HCQ) in sarcoidosis-associated uveitis (SAU) and idiopathic uveitis (IdU).

**Methods:**

This monocentric retrospective study included 42 patients with SAU and 15 patients with IdU treated with HCQ for at least six months between March 2003 and December 2022. All types of uveitis were included. Most patients had chronic bilateral granulomatous SAU or IdU. Efficacy was determined by the success rate of HCQ at 6 and 12 months, and at the last visit and was defined as having control of inflammation, no more than 5 mg prednisone daily and less than or equal to 2 drops of dexamethasone phosphate 0.1%, and no treatment failure due to safety. Biomicroscopic data, best-corrected visual acuity, inflammation grading (SUN criteria) and optionally data from optical coherence tomography and fluorescein or indocyanine green angiography were assessed. The Fisher’s exact test and the Wilcoxon rank test were used for the comparison of qualitative data and quantitative data respectively. Prednisone dose was compared using a mixed model.

**Results:**

The median [IQR] duration to the last visit was 19.5 [11-44.8] months in SAU patients and 18 [13–38] months in IdU patients. At the last visit, 55% of patients with SAU (including 70% of anterior SAU and 77% of intermediate SAU) and 40% patients with IdU (including 27% of anterior IdU) were successfully treated with HCQ ; the median [IQR] prednisone dose decreased from 10 [8.0-27.5] to 4 [2.5–5.75] mg/day and from 15.5 [12.5–19.5] to 3.0 [3.0–5.0] mg/day in SAU and IdU patients, respectively. The reduction in median prednisone dose was significant in patients with SAU (*p* = 0.002). The incidence rate ratio of flare was 0.73 (*p* = 0.143) in SAU patients and 0.26 (*p* < 0.001) in IdU patients.

**Conclusion:**

HCQ could be an interesting therapeutic option for specific types of SAU and IdU. Additionally, HCQ decreased the incidence of flare-ups and the need for oral prednisone in these patients.

**Supplementary Information:**

The online version contains supplementary material available at 10.1186/s12348-026-00598-7.

## Background

Uveitis refers to the inflammation of the iris, ciliary body, vitreous body, retina or choroid. Its incidence is estimated to be 17–52 per 100,000 person-years and its prevalence varies from 38 to 714 per 100,000 persons [1, 2]. Uveitis accounts for 10% to 15% of all cases of total blindness in the United States, defined by the World Health Organization as the best-corrected visual acuity in the better eye of less than 20/400 [3]. It is the fifth most common cause of blindness in people between the ages of 20 and 60 in developed countries [4].

The literature reports 80 causes of uveitis, which can be categorized into five main groups: ophthalmologic entities, infectious diseases, systemic diseases, drug-induced, and unknown origin [5]. Among the patients who are referred to a tertiary centre for uveitis, sarcoidosis is usually found in 2 to 17% of them [6, 7]. Idiopathic uveitis (IdU) represents 23–44% of uveitis cases in Western countries [8–13]. In fact, IdU is typically managed as inflammatory uveitis associated with a known nosological framework, considering the patient’s phenotype, despite the absence of established guidelines. Some studies have suggested that IdU may initially present with a sarcoid-like phenotype, with six to 10% of cases eventually leading to a subsequent diagnosis of sarcoidosis [14, 15].

Topical steroids, systemic glucocorticoids, immunosuppressants such as methotrexate, or biologics such as anti-tumor necrosis factor (TNF) are part of the therapeutic arsenal in autoimmune uveitis. However, side effects and non-response remain high with these treatments [16, 17]. Indeed, a post-hoc analysis of VISUAL I revealed that the incidence of adverse events related to corticosteroids was 12.6 times higher during treatment than after discontinuation [16]. In addition, infectious adverse events related to anti-TNF were reported in 65% of cases in VISUAL III study [18].

Hydroxychloroquine (HCQ), an antimalarial drug, has demonstrated immunomodulatory effects in several systemic diseases, including sarcoidosis. In the latter, its efficacy has been reported in treating various organ involvement, such as skin, joints, lungs, and neurosarcoidosis, as well as hypercalcemia [19–23].

In an experimental murine model of autoimmune uveitis, Hu et al. recently demonstrated that HCQ reduces the expression of inflammatory and chemokine factors by inhibiting the activation of naive CD4 + T cells and promoting T regulatory cells [24].

More recently, our team introduced the use of HCQ in the treatment of 27 patients with sarcoid uveitis despite the potential ocular toxicity of antimalarials [25]. In this retrospective study with a mean duration of HCQ of 20 months, efficacy seemed to be more notable in anterior and intermediate uveitis. Other benefits appeared to be a significant reduction in systemic corticosteroid therapy and in the number of relapses. However, HCQ was discontinued in 12 patients during follow-up, including 8 patients because of ineffectiveness. The aim of the study was to confirm the potential beneficial effect of HCQ treatment in a larger cohort of patients with sarcoidosis-associated uveitis (SAU) and to evaluate the efficacy and safety of HCQ in the treatment of IdU.

## Methods

### Patients

We retrospectively identified all cases of uveitis among patients seen at the Ophthalmology and Internal Medicine Departments of the Croix Rousse Hospital in Lyon, France, between March 2003 and December 2022. The diagnosis of uveitis was confirmed in all patients by an ophthalmologic assessment and a complete and systematic clinical examination by an internist. Uveitis was classified according to the Standardization of Uveitis Nomenclature (SUN) [26]. Acute or chronic status was recorded in accordance with the SUN definition.

Patients were eligible for inclusion if they were diagnosed with SAU or IdU according to a standard screening protocol [15, 27, 28], had received HCQ treatment for at least 6 months, and were not receiving immunosuppressive therapy, aside from oral corticosteroids, at the start of HCQ. Eighteen patients from our previous study were included [25] with an extended follow-up of 26 months [IQR 1.5–36]. Patients treated for extraocular involvement were excluded if their uveitis was inactive at the initiation of HCQ.

HCQ treatment was administered for a minimum duration of 6 months at a dose of 200 mg twice daily or 200 mg once daily for patients with a body weight less than 50 kg. Although the corticosteroid tapering schedule was not standardised, the initial dose ranged from 0.5 to 1 mg/kg, with a target dose of 15 to 20 mg at month 3, 5 to 10 mg at month 6, and ≤ 5 mg at month 12.

All patients underwent a standardized screening protocol for uveitis, which encompassed a complete blood count (CBC), a C-reactive protein (CRP), an erythrocyte sedimentation rate (ESR), a serological test for syphilis, a tuberculin skin test and a chest X-ray [27]. Human leukocyte antigen (HLA)-B27 typing was conducted in cases of acute anterior uveitis. In granulomatous uveitis or chronic anterior uveitis, angiotensin-converting enzyme (ACE) levels and a chest computed tomography (CT) scan were performed [27, 28]. The screening protocol for sarcoidosis included conjunctival or cutaneous biopsies when clinical signs were indicative. Certain patients also underwent a minor salivary gland biopsy (MSGB), transbronchial lung biopsy, bronchoalveolar lavage (BAL), cerebral magnetic resonance imaging (MRI), or nuclear imaging. In select cases, this work-up included an anterior chamber paracentesis (with polymerase chain reaction testing for Herpesvirus, *Toxoplasma*, or 16 S rRNA, and occasionally interleukin-10 quantification), a vitreous biopsy, and/or lumbar puncture, when deemed necessary [15, 27, 28].

Biopsy-proven sarcoidosis was defined as histological demonstration of non-caseating granuloma and exclusion of other diseases, according to the World Association for Sarcoidosis and Other Granulomatous Disorders/American Thoracic Society/European Respiratory Society (WASOG/ATS/ERS) criteria [29, 30]. In the absence of histological evidence, Abad’s modified criteria were used. Patients were classified as presumed SAU if they had at least two of the following criteria: (1) typical changes on chest X-ray or CT scan, (2) a predominant CD4 lymphocytosis on BAL fluid analysis, (3) an elevated serum angiotensin-converting enzyme (sACE) and (4) an ^18^F-fluorodeoxyglucose (^18^F-FDG) uptake on positron emission tomography (PET) instead of 99mTc scintigraphy. Patients with only one of the above criteria were classified as probable SAU [31]. Moreover, we used the SUN and IWOS criteria for SAU [29, 32].

The study received approval from the local ethics committee in February 2019 (N° 19–31) and was registered on clinicaltrials.gov (NCT 038663782-Lyon sarcoid uveitis cohort) and (NCT 03877575-Lyon uveitis study).

### Data collection

The following ophthalmic examination characteristics were collected at baseline, 3, 6, 12 months, and last follow-up visit available on HCQ, if available: biomicroscopic evaluation (conjunctiva, cornea, anterior chamber, iris, lens, vitreous, and retina), intraocular pressure (IOP), best-corrected visual acuity (BCVA), and optionally optical coherence tomography (OCT) and fluorescein and/or indocyanine green angiography. Additionally, we reported the presence or absence of granulomatous uveitis. LogMAR BCVA was calculated from visual acuity according to the Monoyer chart used in France, a standard chart for measuring visual acuity that employs a series of letters or symbols at specific sizes to assess visual acuity [33]. Ocular complications such as cataract, glaucoma, macular edema, papilledema, retinal vasculitis, optic atrophy, multifocal choroiditis, and synechiae were collected. Patient demographics, extraocular manifestations, and all previous or concomitant therapies, including topical steroid injections, were also recorded. Macular toxicity was determined if the patient underwent an examination that included visual acuity testing, colour vision testing, OCT and/or electroretinography (ERG). This screening was not conducted systematically.

HCQ success was determined at 6 months, 12 months, and final visit and was defined by: (1) ≤ 0.5 + anterior chamber cells, ≤ 0.5 + vitreous cells, ≤ 0.5 + vitreous haze, and no active retinal/choroidal lesions, (2) ≤ 5 mg oral prednisone daily and ≤ 2 drops dexamethasone phosphate 0.1% (or equivalent) daily, and (3) no discontinuation of HCQ due to adverse effects [25].

Flares were evaluated prior to and during the HCQ period. Flare was characterized by the presence of reactivation of ocular inflammation, including: anterior chamber cells ≥ 1+, or vitreous haze ≥ 1+, or active chorioretinal lesions, or inflammatory retinal vascular lesions, or optic nerve inflammation, or by chart review.

Relapses were assessed at 6 months, 12 months, and the final visit and defined as a flare requiring a modification in the therapeutic regimen (intraocular corticoid injection, increase in corticosteroid dose, or change in steroid-sparing agent).

The median dose and the number of patients requiring systemic corticosteroids were compared before HCQ initiation, at 3, 6, and 12 months and at the last visit. Corticosteroid dependence was defined as the inability to control ocular inflammation with > 5 mg of oral prednisone or > 2 drops of dexamethasone phosphate 0.1% (or equivalent) [25]. Frequency of ocular complications and HCQ safety were also evaluated.

The main objective was to assess the effectiveness of HCQ after 6 and 12 months, and at the final visit based on the number of successes achieved. Secondary outcomes assessed the frequency of relapses at 6, 12 months, and at the final visit; the time before the first flare; the incidence rate of flare prior and during HCQ treatment; corticosteroid dependence; impact on logMAR; and the safety profile of HCQ.

### Statistical analysis

All patients who fulfilled Abad’s criteria were included in the statistical analysis. Means and standard deviations or median and interquartile range [IQR] were used for quantitative data and frequencies and percentages for qualitative data. Henry’s line and the Shapiro-Wilk test were used to assess the normality of the distribution. Qualitative data were compared with the Fisher’s exact test and quantitative data with the Wilcoxon rank test. Prednisone dose and BCVA were compared using a mixed model. This model was not applicable for the analysis of the prednisone in IdU patient due to the sample size. Poisson regression was used for the comparison of the incidence rate ratio. Statistical significance was set at a p-value < 0.05. Analyses were performed using version 4.3.1 of R (R Core Team (2023). R: A Language and Environment for Statistical Computing. R Foundation for Statistical Computing, Vienna, Austria. https://www.R-project.org/*).*

## Results

### Characteristics of patients

**Fig. 1:**
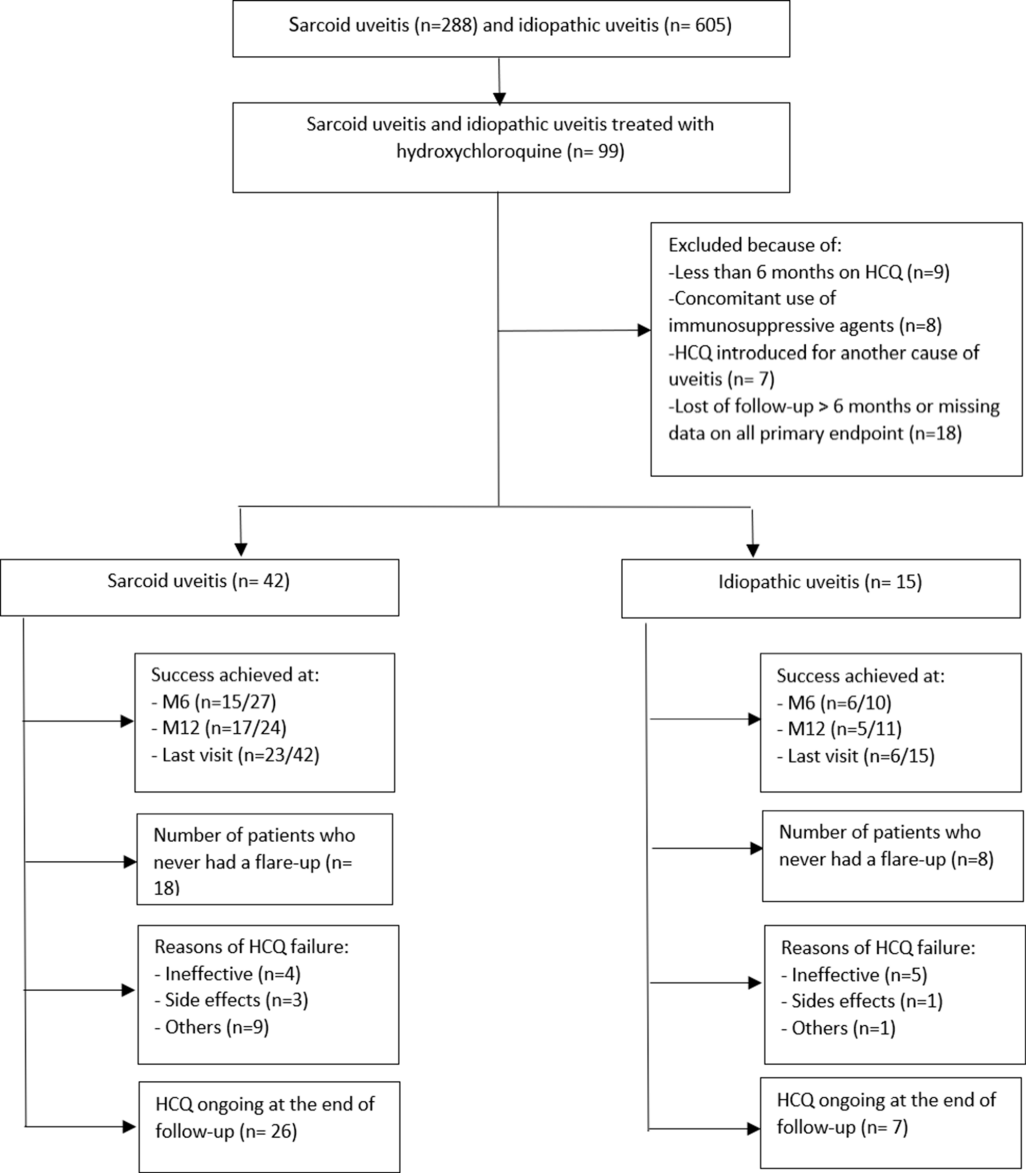
Flow chart

Among the 1868 patients with uveitis treated at our university hospital between March 2003 and December 2022, 99 patients received treatment with HCQ. Among them, 8 patients were excluded due to concomitant immunosuppressive therapy, 9 patients were treated with HCQ for less than 6 months, 7 patients were treated with HCQ for extraocular manifestations of sarcoidosis, and 18 patients were lost to follow-up (Fig. 1).

Fifty-seven patients were included in this study, including 42 patients with SAU and 15 patients with IdU. Their characteristics at diagnosis are detailed in Tables 1 and 2. The overall mean age at diagnosis was 51.2 years (± 17.9). Thirty-one patients (54%) were female. Thirty-nine (68%) were Caucasian, 11 (19%) were North African, and 7 (12%) were Afro-Caribbean.

**Table 1:** Epidemiologic data and clinical features of patients at baseline

Number of patients *n* (%)	Sarcoid uveitis	Idiopathic uveitis	*p*-value
	42 (74)	15 (26)	
Mean age (year) ± SD	52.8 ± 17.4	46.3 ± 19.0	0.28
Sex n (%)			
Female	23 (55)	8 (53)	1
Male	19 (45)	7 (47)	1
Ethnic groups n (%)			
Caucasian	28 (76)	11 (73)	0.75
North African	8 (19)	3 (20)	1
Afro Caribbean	6 (14)	1 (7)	0.66
Sarcoidosis probability, n (%)			
Proven	22 (52)	NA	
Presumed	14 (33)	NA	
Probable	6 (14)	NA	
Systemic features, n (%)	29 (69)	0	
Thoracic	26 (62)	0	
Cutaneous	6 (14)	0	
Joint	2 (5)	0	
ENT	4 (10)	0	
Digestive	1 (2)	0	
Lymph nodes	3 (7)	0	
Nervous system	1 (2)	0	
Uveitis characteristics, n (%)			
Bilateral	32 (76)	8 (53)	0.11
Chronic	26 (62)	10 (67)	1
Granulomatous	32 (76)	10 (67)	0.51
Localization			
Anterior	7 (17)	5 (33)	0.27
Intermediate	6 (14)	0	0.32
Posterior	2 (5)	1 (7)	1
Anterior + Intermediate	3 (7)	6 (40)	0.07
Intermediate + Posterior	3 (7)	0	0.56
Panuveitis	21 (50)	3 (20)	0.067
Grade of anterior chamber cells ≥ 0,5 ≥ 1 ≥ 2	10 (24) 12 (29) 6 (14)	5 (33) 5 (33) 3 (20)	0.507 0.75 0.69
Grade of vitreous inflammation ≥ 0,5 ≥ 1 ≥ 2	10 (24) 12 (29) 5 (12)	3 (20) 4 (27) 2 (13)	1 1 1
Glaucoma	12 (29)	7 (47)	0.22
Synechiae	11 (26)	5 (33)	0.74
Macular edema	16 (38)	5 (33)	1
Papillary edema	10 (24)	6 (40)	0.32
Retinal vasculitis	14 (33)	4 (27)	0.75
Multifocal choroiditis	6 (14)	5 (33)	0.14
Cataract	7 (17)	7 (47)	0.03
Optic atrophy	4 (10)	0	0.56
Median LogMAR BCVA [IQR]	0.0 [0.0-0.3]	0.0 [0.0-0.2]	0.54
Median duration of follow-up before HCQ (month)[IQR]	20 [6–52]	27 [18–52]	0.36
Previous treatment, n (%)			
Oral prednisone	17 (40)	5 (33)	0.76
MTX	5 (12)	0	0.31
AZA	2 (5)	0	1
MMF	1 (2)	2 (13)	0.17
Salazopyrine	0	2 (13)	0.66
Antituberculosis	1 (2)	3 (20)	0.17
Anti herpes simplex	1 (2)	2 (13)	0.17
Median flare before HCQ [IQR]	1 [1–2]	2 [1–4]	0.08

AZA: azathioprine; MMF : mycophenolate mofetil ; BCVA: best-corrected visual acuity; ENT: Ear nose and throat; HCQ: hydroxychloroquine, IQR: interquartile range; MTX: methotrexate; SD: standard deviation ; NA : not applicable

**Table 2:** Associated treatment and reasons of HCQ onset

	Sarcoid uveitis *n*(%)	Idiopathic uveitis *n*(%)	*p*-value
**Associated treatment at HCQ onset**			
Corticosteroid eye drops	9 (21)	4 (27)	0.73
Oral prednisone	22 (52)	5 (33)	0.24
median dose in mg/day (IQR)	10.0 [8.0-27.5]	15 [15.0–16.0]	0.97
Intravitreal dexamethasone implant	9 (21)	4 (27)	0.73
Subconjunctival corticosteroid injection	3 (7)	4 (27)	0.07
Ocular injection of antiVEGF	2 (5)	0	1
**Reasons to start HCQ**			
Uncontrolled inflammation	33 (79)	13 (87)	0.71
Steroids dependance	5 (12)	2 (13)	0.65
Ineffective immunosuppressant	2 (5)	0	1
Steroid side effect	2 (5)	0	1

HCQ : hydroxychloroquine ; IQR: interquartile range ; VEGF : vascular endothelial growth factor

Panuveitis was found in 24 (42%) patients, and bilateral uveitis was observed in most patients (n = 40; 70%). Major complications before HCQ were macular edema (n = 21; 37%), glaucoma (n = 19; 33%), retinal vasculitis involving either veins or arteries (n = 18; 32%). Baseline characteristics were comparable between the SAU and IdU groups, except for cataract and anterior with intermediate involvement, which were significantly more prevalent in the IdU cohort (p = 0.03 and p = 0.007, respectively) (Table 1)”.

Uveitis was the first manifestation of sarcoidosis in 29 (69%) patients. Sarcoidosis was histologically proven in 22 (52%) patients, presumed in 14 (33%), and probable in 6 (14%) (Table 1). The SUN criteria were not retrospectively met in 9 (21%) patients. Twenty-nine (69%) patients had extraocular sarcoidosis and the most common manifestations were: thoracic (*n* = 25; 59%), cutaneous (*n* = 6; 14%), ear nose and throat (ENT) (*n* = 4; 10%).

Prior to HCQ, 18 (31%) patients received intravitreal or subconjunctival steroid injections. Two (4%) patients received anti-vascular endothelial growth factor (anti-VEGF) injections. Twenty-two (39%) patients were previously treated with systemic corticosteroids, 5 (9%) with methotrexate (MTX), 1 (2%) with mycophenolate mofetil (MMF), and 2 (4%) with azathioprine (AZA) and 2 (4%) with salazopyrine. Four (7%) patients received empiric systemic antituberculosis (rifampicin and isoniazid) and 3 (5%) patients received antiviral therapy for herpes simplex infection (valaciclovir) resulting in treatment failure (Table 1).

### Efficacy and safety data

HCQ was started at 400 mg daily in all patients except one patient in the IdU group, who was started at 200 mg due to his weight. The reasons for initiating HCQ are shown in Table 2. Most patients had uncontrolled ocular inflammation (*n* = 33; 79% in SAU group and *n* = 13; 87% in IdU group) and corticosteroid dependence (*n* = 5; 12% in SAU group and *n* = 2; 13% in IdU group) (Table 2).

#### Sarcoid associated uveitis

HCQ was administered to treat active uveitis with extraocular involvement of sarcoidosis in 9 (21%) patients, involving the skin (*n* = 6; 14%), joints (*n* = 2; 5%), and multiplex neuritis (*n* = 1; 2%).

**Fig. 2:**
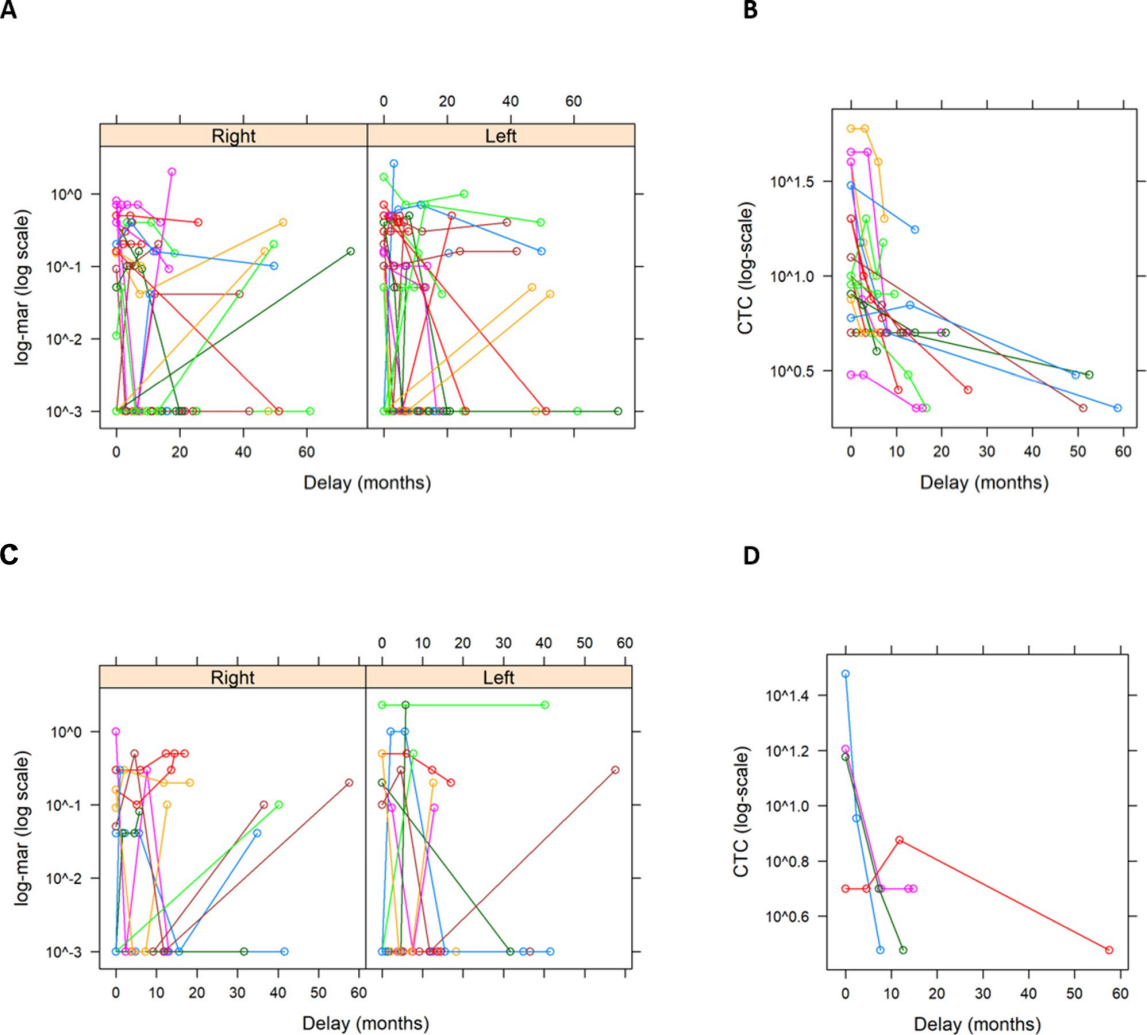
Visual acuity and steroid use outcomes during follow-up. Visual acuity analysis is carried out by a mixed model, incorporating a fixed effect of time, an intercept, and a random slope per patient, as well as random intercepts and slopes per nested eye within the patient level for sarcoid uveitis patient (**A** ; *p* = 0.727 ), and idiopathic uveitis patients (**C** ; *p* = 0.470). Corticosteroid dose analysis is conducted using a mixed model, incorporating a fixed effect of time, an intercept, and a random slope per patient for sarcoid uveitis patient (**B** ; *p* = 0.002). For idiopathic uveitis patients (**D**), no model is adjusted for corticosteroid dosage due to the sample size

Patients were treated with HCQ for a median duration of 24.5 [17.3; 46.5] months. Most of them were also treated with oral prednisone (*n* = 22, 52%) with a median dose of 10 [8.0; 27.5] mg/day in addition to HCQ (Table 2). Among patients on systemic prednisone at baseline, 12 (54%) were able to take ≤ 5 mg/day at the last visit, 6 (27%) required topical injections or implants of corticosteroid during follow-up. The median prednisone dose was reduced from 10 [8.0; 27.5] mg/day at baseline to 4 [2.5; 5.75] mg/day at the last HCQ visit. The reduction in the median prednisone dose was significant in patients with SAU (*p* = 0.002) (Table 3; Fig. 2).

**Table 3:** HCQ outcomes in sarcoid uveitis

	Total population (*N* = 42)	AU (*n* = 7)	IU (*n* = 6)	PU (*n* = 2)	AU + IU (*n* = 3)	IU + PU (*n* = 3)	*P* (*n* = 21)
Median duration of HCQ, months [IQR]	24.5 [17.25–46.5]	36 [24-52.5]	47.5 [36.8–51.5]	45.5 [41.8–49.2]	23 [15.0-40.5]	26 [18.5–33.5]	19 [14–29]
Median duration to the last visit on HCQ, months [IQR]	19.5 [11-44.8]	20 [17.5–49]	44 [35.8–49.3]	43.5 [40.8–46.3]	20 [13.5–36]	25 [18–33]	14 [9–20]
**Primary outcome**							
HCQ success, n (%)							
M6 (*n* = 27) M12 (*n* = 24) Last visit (*n* = 42)	15 (56) 17 (71) 23 (55)	5 4 5	0 0 6	0 0 0	0 1 2	1 2 1	9 10 9
**Secondary outcomes**							
Relapse, n (%)							
M6 (*n* = 27) M12 (*n* = 24) Last visit (*n* = 42)	7 (26) 7 (29) 10 (24)	0 0 1	2 2 0	1 2 1	1 0 1	1 0 1	2 3 6
Median variation of							
LogMar [IQR]							
M0 M3 M6 M12 Last visit	0.00 [0.00- 0.20] 0.00 [0.00- 0.16] 0.00 [0.01–0.18] 0.00 [0.00- 0.16] 0.00 [0.00- 0.15]						
Reasons of HCQ failure, n (%) Ineffective Adverse effect Other reasons	16 (38) 4 (10) 3 (7) 9 (21)	0 1 0	0 1 1	0 0 1	0 0 1	1 1 1	3 0 5
Time to first flare (months) [IQR]	13 [8–18]	15 [12–20]	35 [27–40]	16 [15–16]	11 [9–13]	5 [5–5]	12 [8–14]
Incidence rate of flare before vs. on HCQ (100 p/y)	52 vs. 42	109 vs. 43	29 vs. 43	200 vs. 55	37 vs. 46	72 vs. 31	50 vs. 39
Median dose Prednisone (mg)[IQR]							
M3 M6 M12 Last visit	7.5 [5.0–15.0] 5 [5.0-7.5] 5 [2.5-5.0] 4 [2.5–5.8]						
Prednisone dependence at last visit, n(%)	4 (10)	0	0	0	1	0	3
Eye drops dependence at last visit, n(%)	2 (5)	1	0	0	0	0	1

AU: anterior uveitis; HCQ: hydroxychloroquine; IU: intermediate uveitis; PU: posterior uveitis ; P : panuveitis; p/y: person-years ; NA : not applicable ; IQR : interquartile range

All results at M6 and M12 should be interpreted cautiously due to missing data

The number of relapses was 7/27 (26%) at 6 months, 7/24 (29%) at 12 months, and 10/42 (24%) at the last visit on HCQ. In the subgroup analysis, 20% of patients with anterior SAU (including both isolated anterior SAU and anterior-associated intermediate SAU) and 11% of patients with intermediate SAU (including both isolated intermediate SAU and intermediate-associated posterior SAU) experienced relapses at the last visit. The incidence rate ratio of flare was 0.73 (*p* = 0.143; [95% CI: 0.47, 1.10]), and the median time to first flare was 13 [8; 18] months. The most common reason for being classified as relapse was macular edema. Eighteen (42%) patients never had flare. Of the 24 patients who experienced a flare, two were on 200 mg HCQ after achieving success at 12 months and two were non-compliant. Seventeen (71%) of these required local treatment, including: dexamethasone eye drops (*n* = 1, 4%), subconjunctival corticosteroid injections (*n* = 5, 21%), intravitreal dexamethasone implants (*n* = 8, 33%), intravitreal anti-VEGF injections (*n* = 2, 8%), and subtenon corticosteroid injection (*n* = 1, 4%). The others required methotrexate (*n* = 3, 13%) and azathioprine (*n* = 1, 4%) for disease progression, and anti-TNF for corticosteroid sparing (*n* = 1, 4%).

BCVA could be analysed in 82 eyes at baseline, 48 at 3 months, 46 at 6 months, 34 at 12 months and 69 at the last visit. The median variation in logMAR [IQR] was stable from 0.00 [0.00-0.20] to 0.00 [0.00- 0.15]. All changes in logMAR were not statistically significant on HCQ (*p* = 0.727) (Fig. 2). Nine (21%) patients developed cataract and 2 (5%) developed optic atrophy during follow-up. Compared to baseline, macular edema was resolved in 12/16 (75%) patients, papilledema in 9/10 (90%) patients, retinal vein or arterial vasculitis in 14/14 (100%) patients, and multifocal choroiditis in 6/6 (100%) patients at the last HCQ visit. Most of them had received concomitant systemic steroids or subconjunctival or intravitreal steroid injections.

HCQ success was achieved in 15/27 (46%) patients at 6 months, in 17/24 (71%) patients at 12 months, and in 23/42 (55%) patients at the last visit on HCQ. Among patients who achieved success, 77% (10 out of 13) of patients initially on prednisone were taking ≤ 5 mg/day at the last visit on HCQ. In the subgroup analysis, 70% of patients with anterior SAU (including both isolated anterior SAU and anterior-intermediate associated SAU) and 77% of patients with intermediate SAU (including both isolated intermediate SAU and intermediate-posterior associated SAU) experienced success at the last visit.

At the end of follow-up, 26 (62%) patients were on HCQ, including 3 patients who were able to reduce the HCQ dose to 200 mg for effectiveness. Five (12%) patients were able to discontinue prednisone, 4 (10%) were dependent on systemic prednisone, and 2 (5%) were dependent on dexamethasone eye drops (Table 3). HCQ was discontinued in 16 (38%) patients including 4 patients for ineffectiveness, 3 patients for adverse events and 5 of them due to extraocular disease progression. All reasons are detailed in Tables 3 and 5. Two of the 16 patients who had an ERG and/or OCT developed macular toxicity leading to the discontinuation of HCQ. Among them, one patient was treated with HCQ for 18 months while the other received treatment for 41 months.

**Table 4:** Safety results on HCQ

	Sarcoid uveitis *n* (%)	Idiopathic uveitis *n* (%)
Any adverse event	7 (17)	1 (7)
Life-threatening adverse event	0	0
Death	0	0
Adverse event		
* Gastro-intestinal* * Cutaneous lesion* * Cardiac* * Neurologic*,* psychiatric* * Ocular toxicity* * Other*	1 (2) 3 (7) 0 1 (2) 2 (5) 0	0 0 0 0 0 1 (7)

Retrospectively, among the 79% of SAU patients who met the SUN criteria, 18 (43%) achieved success, 7 (17%) experienced a relapse, and 1 (2%) remained dependent on oral steroids at the last visit. The remaining patients had inactive uveitis. Similarly, 83% of SAU patients met the IWOS criteria. Among them, 19 (45%) achieved success, 9 (21%) experienced a relapse, and 1 (2%) remained dependent on oral steroids at the last visit. The remaining patients had inactive uveitis.

Disease progression was observed with thoracic (*n* = 8; 19%), skin (*n* = 2; 5%), joint (*n* = 1; 2%), hepatosplenic (*n* = 8; 19%), lymph node (*n* = 5; 12%), heart (*n* = 1; 2%), nervous system (*n* = 1; 2%) involvement and hypercalcemia (*n* = 1; 2%).

#### Idiopathic uveitis

Patients were treated with HCQ for a median duration of 31.0 [16.5; 40.0] months. Concomitant steroids treatments are described in Table 2. The median prednisone dose was reduced from 15.5 [12.5; 19.5] mg/day at baseline to 3.0 [3.0; 5.0] mg/day at the last HCQ visit (Table 4; Fig. 2). Due to the sample size, no model could be adjusted to assess this reduction. All patients on prednisone at baseline were able to be on ≤ 5 mg/day at 6 months.

**Table 5:** HCQ outcomes in idiopathic uveitis

	Total population *N* = 15	AU (*n* = 5)	IU (*n* = 0)	PU (*n* = 1)	AU + IU (*n* = 6)	IU + PU (*n* = 0)	*P* (*n* = 3)
Median duration of HCQ (months) [IQR]	31.0 [16.5–40.0]	36 [31–36]	NA	59	16 [12.5–21.8]	NA	44 [29.5–52.5]
Median duration to the last visit on HCQ, months [IQR]	18 [13–38]	31 [16–36]	NA	41	12.5 [6.5–17]	NA	40 [27-48.5]
**Primary outcome**							
HCQ success, n (%)							
M6 (*n* = 10) M12 (*n* = 11) Last visit (*n* = 15)	6 (60) 5 (45) 6 (40)	3 1 2	NA NA NA	1 0 1	1 4 1	NA NA NA	1 0 2
**Secondaries outcomes**							
Relapse, n (%)							
M6 (*n* = 10) M12 (*n* = 11) Last visit (*n* = 15)	1 (10) 3 (25) 7 (47)	1 2 2	NA NA NA	0 0 0	0 0 5	NA NA NA	0 1 1
Median variation of LogMar [IQR]							
M0 M3 M6 M12 Last visit	0.00 [0.00-0.16] 0.00 [0,00-0.05] 0.02 [0.00-0.30] 0.00 [0.00-0.13] 0.06 [0.00-0.20]						
Reasons of HCQ failure, n (%)	7 (47)						
Ineffective Adverse effect Other reasons	5 (33) 1 (7) 2 (13)	2 0 2	NA NA NA	0 0 0	2 1 0	NA NA NA	1 0 0
Time to first flare (months) [IQR]	11 [7 − 6]	11 [9–13]	NA	NA	14 [12–18]	NA	4 [4–4]
Incidence rate of flare before vs. on HCQ (100 p/y)	82 vs. 46	169 vs. 59	NA	54 vs. 0	67 vs. 34	NA	47 vs. 30
Median dose Prednisone (mg) [IQR]							
M3 M6 M12 Last visit	10 [9.0–10.0] 5 [5.0–5.0] 5 [3.0- 5.1] 3 [3.0; -5]						
Prednisone dependence at last visit, n(%)	2 (13)	1	NA	0	0	NA	0
Eye drops dependence at last visit, n(%)	1 (7)	0	NA	0	1	NA	0

AU: anterior uveitis; HCQ: hydroxychloroquine; IU: intermediate uveitis; PU: posterior uveitis ; P : panuveitis; p/y: person-years; NA : not applicable

All results at M6 and M12 should be interpreted cautiously due to missing data

The number of relapses was 1/10 (10%) at 6 months, 3/11 (27%) at 12 months, and 7/15 (47%) at the last visit on HCQ (Table 4). In the subgroup analysis, 64% of patients with anterior IdU (including both isolated anterior IdU and anterior-intermediate associated IdU) experienced relapses at the last visit. The incidence rate ratio of flare was 0.26 (*p* < 0.001; [95% CI: 0.14, 0.45]) and the median time to the first flare was 11 [7; 16] months. Eight (53%) patients never had flare. Of the 7 patients who experienced flares, they required dexamethasone eye drops (*n* = 1; 14%), subconjunctival corticosteroid injections (*n* = 3; 43%), intravitreal dexamethasone implants (*n* = 2; 29%) and methotrexate (*n* = 1; 14%).

BCVA could be analysed in 30 eyes at baseline, 12 at 3 months, 20 at 6 and 12 months and 28 at the final visit. The median variation of logMAR [IQR] increased from 0.00 [0.00-0.16] to 0.06 [0.00-0.20]. All changes in logMAR were not statistically significant (*p* = 0.470) (Fig. 2).

One (7%) patient developed optic atrophy. Compared to baseline, macular edema was normalised in 3/5 (60%) patients, papilledema in 6/6 (100%) patients, retinal vein or arterial vasculitis in 3/4 (75%) patients, and multifocal choroiditis in 5/5 (100%) patients at the last HCQ visit. Most of them had received concomitant systemic steroids or subconjunctival or intravitreal steroid injections.

HCQ success was achieved in 6/10 (60%) patients at 6 months, in 5/11 (45%) patients at 12 months and in 6/15 (40%) patients at the last visit on HCQ. In the subgroup analysis, 27% of patients with anterior IdU (including both isolated anterior IdU and anterior-intermediate associated IdU) experienced success at the last visit. On the other hand, 40% of isolated anterior IdU patients achieved success at the last visit (Table 4).

At the end of follow-up, 7 (47%) patients were on HCQ, including one patient who was able to reduce the HCQ dose to 200 mg after achieving success. Three (20%) were on ≤ 5 mg of prednisone, 2 (13%) were dependent on oral prednisone, and 1 (7%) was dependent on dexamethasone eye drops (Table 4).

HCQ was discontinued in 8 (53%) patients due to disease control (*n* = 1; 7%), ineffectiveness (*n* = 5; 33%), non-severe adverse events (*n* = 1; 7%) and progression with myelitis (*n* = 1; 7%) which required methotrexate (Table 4). Adverse events experienced by the patients are detailed in Table 5. Of the 9 patients who had an ERG and/or OCT, none developed macular toxicity.

## Discussion

This study suggests that HCQ can control ocular inflammation, preserve visual acuity, decrease the median prednisone dose required, and reduce the incidence rate of flares in certain types of SAU and IdU.

HCQ is one of the drugs currently used in the treatment of extraocular sarcoidosis [19–22], but only a few studies have evaluated its efficacy for uveitis treatment. Bert et al., have reported the efficacy of HCQ in mild anterior and intermediate sarcoid uveitis [25]. This study also shows that even in a larger and longer cohort, the success rate in SAU patients remains high (*n* = 23, 55%) and that HCQ delays the onset of relapse in these patients. Moreover, subgroup data suggest that patients with the anterior type of IdU have a higher response rate to HCQ compared to other types.

SAU pathogenesis involves several key mechanisms. In addition to genetic susceptibility and potential infectious triggers, the inflammatory process is central to the development of sarcoidosis. Toll-like receptors 2 and 4 (TLR2, TLR 4), expressed by macrophages and dendritic cells, are activated in response to eye antigens or cross-reactive antigens [34, 35]. This activation leads to the stimulation of T cells. Subsequently, pro-inflammatory cytokines such as IL-1, IL-12 and TNF-α are released. This cascade of events results in the differentiation of autoreactive CD4^+^ T cells into Th1 and Th17 subsets expressing INF-γ and IL-2 and IL-17, respectively. These cytokines are responsible for ongoing inflammation and are also involved in granuloma formation. In addition, T reg cell deficiency and serum amyloid A protein secreted by macrophages play a role in granuloma proliferation [34]. The pathogenesis of idiopathic uveitis is not well established but seems to share also a CD4^+^ T cell-mediated inflammatory process involving a possible Th1, and less likely, a Th2 response [36–38].

In an experimental autoimmune uveitis murine model, Hu et al. demonstrated that HCQ suppressed the expression of inflammatory genes producing *ll17a*,* Ifng*,* and Il1b* and decreased the expression of chemokines involved in the recruitment of lymphocytes. In addition, HCQ treatment inhibited the activation and differentiation of CD4^+^ T cells in T effectors Th1 and Th17 and their proliferation. In addition, they demonstrated that HCQ reduced retinal inflammation in mice through photographic and histopathological assessment [24].

Our study presents new insights on HCQ in the treatment of IdU. In the literature, it has been found that IdU shares similarities with the most common causes of uveitis, such as sarcoidosis and HLA B27-related uveitis [14, 15]. This led us to consider some of our patients with IdU as having a sarcoidosis-like phenotype. Our patients with IdU were mainly Caucasian females with a median age of 46 years with chronic bilateral anterior or anterior and intermediate granulomatous uveitis. This demographic similarity is supported by Choi et al. who identified presumed sarcoidosis *(based on IWOS criteria)* in 36.5% of the 52 patients who were diagnosed from 179 patients with IdU. Their patients were mainly Caucasian females with a mean age of 55 years with bilateral intermediate followed by anterior IdU [14]. Additionally, in Richard-Colmant et al. study, 335 patients with IdU were included, and after one year of follow-up, 20 patients received a final diagnosis. Among them, 30% had probable (according to Abad et al. modified criteria) or proven sarcoidosis including 20% diagnosed after undergoing a ^18^F-FDG-PET [15]. Their characteristics were similar to those of the patients in Choi et al. cohort, except their mean age was 52 years and panuveitis was most common. Overall, although our patients with IdU were younger, this supported the idea that they may have a sarcoidosis-like phenotype, characterized by bilateral granulomatous uveitis, justified their inclusion in the study. This decision was based on the assumption that a phenotype-oriented approach could support a similar therapeutic management strategy.

In our study, although patients with IdU appeared to have similar ocular characteristics to those with SAU, there were few differences in their outcomes. First, the reduction in the median prednisone dose in patients with IdU could not be statistically evaluated due to our small sample size. Furthermore, we observed a higher prevalence of the anterior-intermediate associated IdU subgroup. This can be explained by several factors, including the natural evolution of uveitis or its diagnosis at an earlier stage in our tertiary center, variations in the definition criteria for uveitis found in the literature, and the small sample size. This subgroup initially responded to HCQ at 6 and 12 months but experienced relapses at the last visit. Interestingly, they exhibited a reduction in the flare incidence rate ratio, which contradicted the elevated number of relapses observed. In fact, 67% of them experienced their first flare at the last visit. This raises the hypothesis of HCQ resistance in some cases of IdU or its inability to sustain long-term remission. It is widely recognized that intermediate and posterior uveitis can cause complications leading to a poor visual prognosis. However, this should be interpreted with caution due to the absence of an isolated intermediate IdU group for comparison, and the small sample size. Additionally, there is insufficient data on the pathogenesis or potential mechanism of HCQ resistance in uveitis to conclusively support this hypothesis. Furthermore, the response rate was lower among patients in the anterior IdU subgroup when compared to those in the anterior SAU subgroup. Owing to our limited sample size, we were unable to assess complications such as macular edema, papillitis, or retinal vasculitis as predictive factors for relapse or HCQ resistance. Moreover, the study by Prete et al. found that most treatments required to manage IdU included MTX, MMF, cyclosporine A, adalimumab or cyclophosphamide. This resulted in an overall rate of inactive uveitis around 50% at 6 months and approximately 65% at 12 months [39]. Thus, a more comprehensive study is needed to determine confounding factors or predictive risk factors for relapses in IdU. Further studies should be conducted to compare immunosuppressants to HCQ before considering the latter as a first-line steroid-sparing treatment for IdU.

Concerning our SAU patients, the response rates of 71% at 12 months and 55% at the last follow-up visit (median follow-up duration: 18 months) are consistent with those reported in the study by Leclercq et al. [40]. In their cohort of non-anterior SAU patients, a complete or partial response at 12 months was achieved in 60%, 27%, and 43% for those treated with MTX, MMF, and AZA, respectively, with no significant difference between treatment groups. Regarding the event-free survival, they reported a median time of 34.5, 8.4, and 16 months with MTX, MMF, and AZA treatment, respectively, with a significant difference for the MTX group (*p* = 0.02) [40]. In our study, the median time to the first flare was 13 months in SAU patients, suggesting that HCQ may represent a promising therapeutic option that warrants further comparative investigation.

In this study, where most patients were treated with concomitant topical or systemic corticosteroids, we observed an improvement in macular edema, papilledema, retinal vasculitis, and multifocal choroiditis in both the SAU and IdU groups on HCQ. In fact, current guidelines recommend the use of corticosteroids as first-line therapy for these complications [6, 41–43]. Furthermore, some patients treated or not with systemic prednisone at the onset of HCQ, received additional treatment with either topical corticosteroid injections or implants (6/22 and 8/20 in the SAU group and 1/4 and 4/9 in the IdU group, respectively). This may have led to an overestimation of the efficacy of HCQ as a steroid-sparing agent in SAU and IdU patients. Using our definition of success, we accepted a potentially lower rate of success. However, it was essential to aim for a prednisone dose of ≤ 5 mg/day, rather than the typical range of ≤ 7.5-10 mg/day, to mitigate the adverse effects of corticosteroids [25].

Preservation of a patient’s visual acuity and quality of life is one of the main goals in uveitis management. Although HCQ did not improve visual acuity, it did help stabilize it, suggesting a positive effect in limiting vision loss. Several factors, including cataract, glaucoma, optic atrophy, and other causes of maculopathy, may have influenced these outcomes. Due to our sample size, we were unable to investigate thoroughly these factors as potential confounders affecting visual acuity. Nevertheless, the role of HCQ in maintaining visual function in patients with uveitis is promising.

The safety profile of HCQ is well established [44]. One concern in uveitis is the risk of developing antimalarial-induced retinal toxicity. Long-term use, cumulative dose of HCQ or pre-existing maculopathy have been shown to increase this risk [45, 46]. In our study, screening for macular toxicity was poor because most patients were on HCQ for less than 2 years. Nevertheless, among the few patients who had ERG, 13% of patients with SAU developed antimalarial-induced maculopathy. Additionally, they were treated with HCQ for less than 4 years, despite the fact that an increased risk of antimalarial toxicity is usually observed after five years of cumulative treatment [46]. Preexisting maculopathy is commonly associated with an increased risk of developing antimalarial-induced maculopathy due to previous tissue damage or difficulty in detecting this complication during screening [46]. One of the two patients had macular edema, but no other ocular factors were identified, and the assessment of other comorbidities was limited by the design of our study. Further and longer-term studies are needed to confirm that HCQ could be a safe therapeutic option without affecting the visual prognosis. In addition, a more frequent screening in this population also needs to be assessed. Moreover, our study did not find any cases of cardiomyopathy associated with the macular toxicity of antimalarial drugs, a phenomenon reported in approximately 12% of patients in a few series [47]. Furthermore, HCQ toxicity does not require blood monitoring as frequently as immunosuppressive agents and can be used during pregnancy [46]. However, the risk of non-adherence to HCQ remains a concern. As reported in a few series of patients with refractory or flaring systemic lupus erythematosus (SLE), non-compliance was observed in 7–18% of cases [48, 49]. Similarly, our two noncompliant SAU patients had blood HCQ concentrations of 0.61 mg/L and 0.15 mg/L at their first flare on HCQ. These levels were below the 1.00 mg/L cutoff commonly used as a predictive biomarker associated with reduced flares in SLE studies [50]. This suggests the importance of measuring HCQ exposure during follow-up, particularly in cases of relapse. This needs to be evaluated in an additional study.

This study has several limitations. First of all, the absence of a control group and the retrospective nature of the study were limitations in assessing the primary outcomes and the corticosteroid-sparing effect. Moreover, due to our limited sample size, comparative analysis of success rates, relapses, and flare rates could not be performed in the SAU and IdU subgroups. In addition, we selected our SAU patients based on Abad’s criteria instead of the SUN criteria established in 2021 [32]. Retrospectively, only 21% ptients did not meet the SUN criteria and 17% the IWOS criteria for SAU, suggesting that Abad’s criteria can identify most SAU patients who also meet the SUN or IWOS criteria. Regarding the comparison with the SUN criteria, which are more restrictive, these differences are explained by: the presence of hilar lymph node uptake on 18F-FDG PET while the CT scan or chest X-ray was considered normal (*n* = 1); the presence of mediastinal uptake on 18F-FDG PET instead of hilar lymphadenopathy on CT scan (*n* = 2); and the presence of extraocular organ uptake on 18F-FDG PET consistent with sarcoidosis (*n* = 2). One patient fulfilled the criteria for presumed SAU according to Abad’s classification, due to elevated ACE serum levels and a CD4/CD8 ratio > 3.5 in BAL fluid. Three patients fulfilled the criteria for probable SAU according to Abad’s classification, due to elevated ACE serum levels. All of them had negative IGRA (data available in the appendix). Notably, 17 of 21 SAU patients who underwent ^18^F-FDG-PET showed hypermetabolic foci consistent with sarcoidosis. Among them, the diagnosis of presumed SAU was made in seven patients and probable SAU in two patients. Few studies have found that using ^18^F-FDG-PET can aid in the classification of 20–30% of cases of IdU or suspected SAU [15, 51]. This finding suggest that ^18^F-FDG-PET should be considered when classifying cases of SAU. Moreover, according to the screening protocol, tuberculosis must be ruled out before establishing a diagnosis of SAU or IdU. Among SAU patients, three had a positive QuantiFERON-TB test, and all of them had histological evidence of sarcoidosis. One patient received treatment for latent tuberculosis infection due to the need for immunosuppressive therapy. This information was unavailable for the other two patients. Although three IdU patients were treated with antituberculosis therapy, none of them responded to “possible ocular TB” criteria regarding the 2015 Gupta classification [52] or the SUN criteria [53]. None of these patients could be at the end of treatment considered as having ocular tuberculosis, as a 6-month course of antituberculosis therapy failed to improve ocular inflammation. That led us to consider them as IdU with a sarcoid-like phenotype [54]. Additionally, we previously found that 16.5% of 1,075 uveitis patients followed at our institution had a positive IGRA test, with 65% of them ultimately receiving a final diagnosis other than tubercular uveitis, including SAU and idiopathic uveitis. Another limitation lies in the substantial amount of missing data, which accounted for 26% and 34% at 6 months, and 43% and 27% at 12 months for SAU and IdU groups, respectively. This was largely due to the lack of systematic follow-up during this time period. In practice, patients with mild uveitis have longer follow-up intervals. Furthermore, it should be noted that the patients evaluated at 6 and 12 months were not consistently the same, making it difficult to interpret treatment success and relapse results during these periods. Nonetheless, it is worth mentioning that the analysis conducted on the last visit did not reveal any significant differences compared to the 6- and 12- month periods. Moreover, prednisone tapering was dependent on the physician’s judgement which may have affected the success rate. Additionally, using the last medical information on HCQ to define the last visit may have affected the success and relapse rates, given the substantial variation in time between HCQ initiation and the last visit among patients.

## Conclusion

HCQ could be a promising therapeutic option for anterior and intermediate SAU and anterior IdU, and a potential corticosteroid-sparing agent. However, longer and randomised controlled trials comparing immunosuppressants, such as MTX, to HCQ are required in these indications to confirm its efficacy, corticosteroid-sparing ability, and safety with regard to retinal toxicity.

## Supplementary Information

Below is the link to the electronic supplementary material.

Supplementary Material 1: Appendix

## Data Availability

The datasets analysed are not publicly available but can be made available from the corresponding author on reasonable request.

## Abbreviations

ATS: American Thoracic Society
AZA: Azathioprine
BAL: Bronchoalveolar lavage
ENT: Ears, Nose and Throat
ERS: European Respiratory Society
HCQ: Hydroxychloroquine
HLA: Human leukocyte antigen
IFN: Interferon
IL: Interleukine
IdU: Idiopathic uveitis
IWOS: International Workshop on Ocular Sarcoidosis
LT CD4^+^: Lymphocyte T CD4^+^
MMF: Mycophenolate mofetil
MTX: Methotrexate
PET: Positron emission tomography
SAU: Sarcoidosis-associated uveitis
SUN: Standardization of Uveitis Nomenclature
TNF: Tumor Necrosis Factor
Treg: Regulatory T cells
WASOG: World Association of Sarcoidosis and other Granulomatous Disorders
^18^F-FDG: ^18^F-fluorodeoxyglucose
