# Supplementary material for: Hydroxychloroquine in the treatment of sarcoidosis-associated uveitis and idiopathic uveitis
Source: J Ophthalmic Inflamm Infect. 2026 May 28;16:37. doi: 10.1186/s12348-026-00598-7 (PMC13407791; doi:10.1186/s12348-026-00598-7)
Supplement: Supplementary file 1 — Supplementary Material 1: Appendix [file 12348_2026_598_MOESM4_ESM.docx]

# Supplementary Appendix

This appendix has been provided by the authors to give readers additional information about their work.

Supplement to: S. Plavonil, R. Jacquot, A,Bert, Y. Jamilloux & Al. Hydroxychloroquine in the treatment of sarcoidosis-associated uveitis and idiopathic uveitis. Journal of Ophthalmic Inflammation and Infection. 2026. https://doi.org/10.1186/s12348-026-00598-7.

# SUPPLEMENTARY APPENDIX

## TABLE OF CONTENT

## Tables ………………………………………………………………………………..3

**Table S6**. Clinical and paraclinical tests results used to classify SAU patients and response to HCQ in patients excluded by the SUN criteria

| Patient | 3 | 8 | 15 | 16 | 26 | 27 | 33 | 38 | 43 |
| --- | --- | --- | --- | --- | --- | --- | --- | --- | --- |
| **Classification by Abad’s criteria** | Presumed | Presumed | Presumed | Probable | Probable | Probable | Probable | Probable | Presumed |
|  |  |  |  |  |  |  |  |  |  |
| **Classification by IWOS criteria * **** | *Excluded* | *Excluded* | *Excluded* | *Excluded* | Possible | Possible | *Excluded* | *Excluded* | Possible |
| *Intraocular clinical signs ⴕ* |  | | | | | | | | |
| 2 oculars signs | + | + | + | + | - | - | + | + | - |
| ≥ 3 oculars signs | - | - | - | - | + | + | - | - | + |
| *Results of systemic investigations* |  |  |  |  |  |  |  |  |  |
| BHL in chest X-ray or CT^$^ | - | - | - | - | - | - | - | - | - |
| Negative tuberculin test or IGRA | + | + | + | + | + | + | + | + | + |
| Elevated serum ACE^$^ | - | + | + | + | + | + | - | - | - |
| Elevated serum lysozyme | ND | + | + | - | + | ND | ND | + | + |
| Elevated CD4/CD8 ratio (>3.5) in BAL fluid^$^ | + | + | - | - | ND | ND | - | ND | ND |
| Abnormal hyperfixation of ^18^F-FDG on PET imaging (or Ga scintigraphy) ^$^  *Localization of uptake* | +  *ENT* | ND | *+*  *ML* | ND | ND | *-* | +  *AL* | +  *HL* | +  *ML* |
| Lymphopenia (<1000/µL) | - | - | - | - | - | - | - | - | - |
| Parenchymal lung changes | - | + | + | - | - | - | + | - | - |
|  |  |  |  |  |  |  |  |  |  |
| Systemic symptoms suggestive of sarcoidosis | - | - | - | - | - | Erythema nodosum | Myelitis | - | - |
| *HCQ success* |  |  |  |  |  |  |  |  |  |
| At M6 | NA | - | - | + | - | + | NA | - | NA |
| At M12 | NA | - | - | + | NA | + | - | - | - |
| At the last visit | + | + | + | + | NR | - | + | NR | + |
| *Relapse* |  |  |  |  |  |  |  |  |  |
| At M6 | NA | - | + | - | + | - | NA | + | NA |
| At M12 | NA | + | + | - | NA | - | + | + | + |
| At the last visit | - | - | - | - | - | + | - | - | + |
| Cause at the time of relapse | NA | Macular edema | Macular edema | NA | Vasculitis | Anterior uveitis | Papillary edema | Macular edema | Macular edema |

**presumed SAU by IWOS criteria include: 2 oculars signs with bilateral hilar lymph nodes without histological proof of ocular sarcoidosis
**possible SAU by IWOS criteria include : at least 3 oculars signs with two paraclinical systemic investigations without histological proof of ocular sarcoidosis
ⴕ intraocular clinical signs used by the Revised IWOS criteria : Mutton-fat keratic precipitates/iris nodules, trabecular meshwork nodules/tent-shaped peripheral anterior synechia, Snowball/string of pearls vitreous opacity, Multiple chorioretinal peripheral lesions, Periphlebitis/macroaneurysm, Optic disc nodule(s)/Choroidal nodule, Bilaterality ;*^$^ paraclinical systemic investigations used by Abad’s criteria
*SAU, sarcoidosis associated uveitis, ACE, angiotensin converting enzyme; BAL, bronchoalveolar lavage; BHL, bilateral hilar lymphadenopathy; ML, mediastinal lymph nodes; ENT, Ear nose and throat; AL, axillar lymph nodes; CT, computed tomography; Ga, gallium-67; IWOS, International Workshop on Ocular Sarcoidosis; ND, not done; PET, positron emission tomography ; IGRA, interferon gamma release assay;* NA, not applicable due to missing data ; NR, not responder to succes due to corticosteroid dose > 5mg/d but inactive.
